# Supplementary material for: JUN mediates the senescence associated secretory phenotype and immune cell recruitment to prevent prostate cancer progression
Source: Mol Cancer. 2024 May 29;23:114. doi: 10.1186/s12943-024-02022-x (PMC11134959; doi:10.1186/s12943-024-02022-x)
Supplement: Supplementary file 1 — Supplementary Material 1. [file 12943_2024_2022_MOESM1_ESM.docx]

**­­­Supplementary Figures**


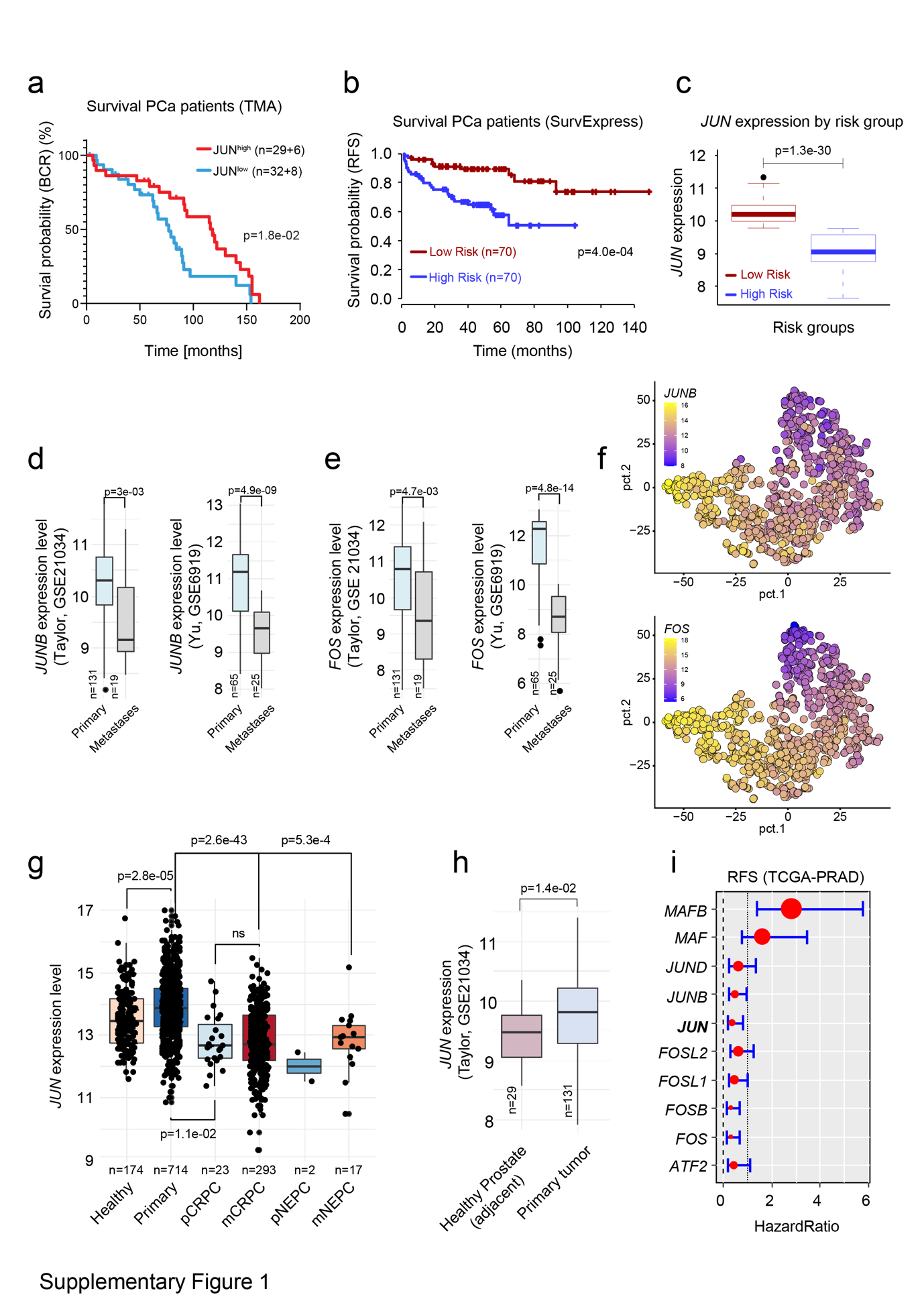


**Supplementary Figure 1: Levels of JUN define progression stages in prostate cancer dependent on PTEN.** a) Kaplan-Meier survival analysis of human prostate tumors (n=75) used for analysis of TMAs stained with an antibody against JUN. Patient groups stratified by presence or absence of JUN show significantly (p=1.8e-02) different biochemical recurrence (BCR)-free survival in (%). b) Kaplan-Meier analysis of relapse-free survival (RFS) of PCa patient RNA-seq data [35] were analysed with the SurvExpress web tool [36]. The groups were stratified according to the prognostic index (PI) into high (red) and low (blue) risk groups. The group comparison was performed with a logrank test (p=4.0e-04). c) *JUN* mRNA expression was surveyed in the individual patient risk groups generated in b). d-e) *JUNB* and *FOS* mRNA levels in prostate tumors comprised in the Taylor [35] (p=3.0e-03 and p=4.7e-03) and Yu datasets [37] significantly (p=4.9e-09 and p=4.8e-14) discriminated primary tumors and metastases. f) Overlay of *FOS* and *JUNB* mRNA expression with the principal component analysis (PCA) from Fig. 1e. *FOS* and *JUNB* levels are color coded from high expression (yellow) to low expression (blue). g) Investigation of *JUN* mRNA expression in normal prostate tissue, primary adenocarcinoma and primary (p) and metastatic (m) CRPC and NEPC. Data were retrieved from [38]. h) *JUN* levels significantly (p=1.4e-02) discriminate healthy adjacent prostate tissue (n=29) and primary (n=131) prostate tumors. Data were retrieved from [35]. i) Representation of hazard ratios (95% confidence interval) of AP-1 family genes determined by Kaplan-Meier analyses with the KMplot tool. The TCGA-PRAD data were used for the analysis [33]. Hazard ratios refer to RFS. In d, e, g, h statistical significance was determined by an unpaired, two-sided t-test.


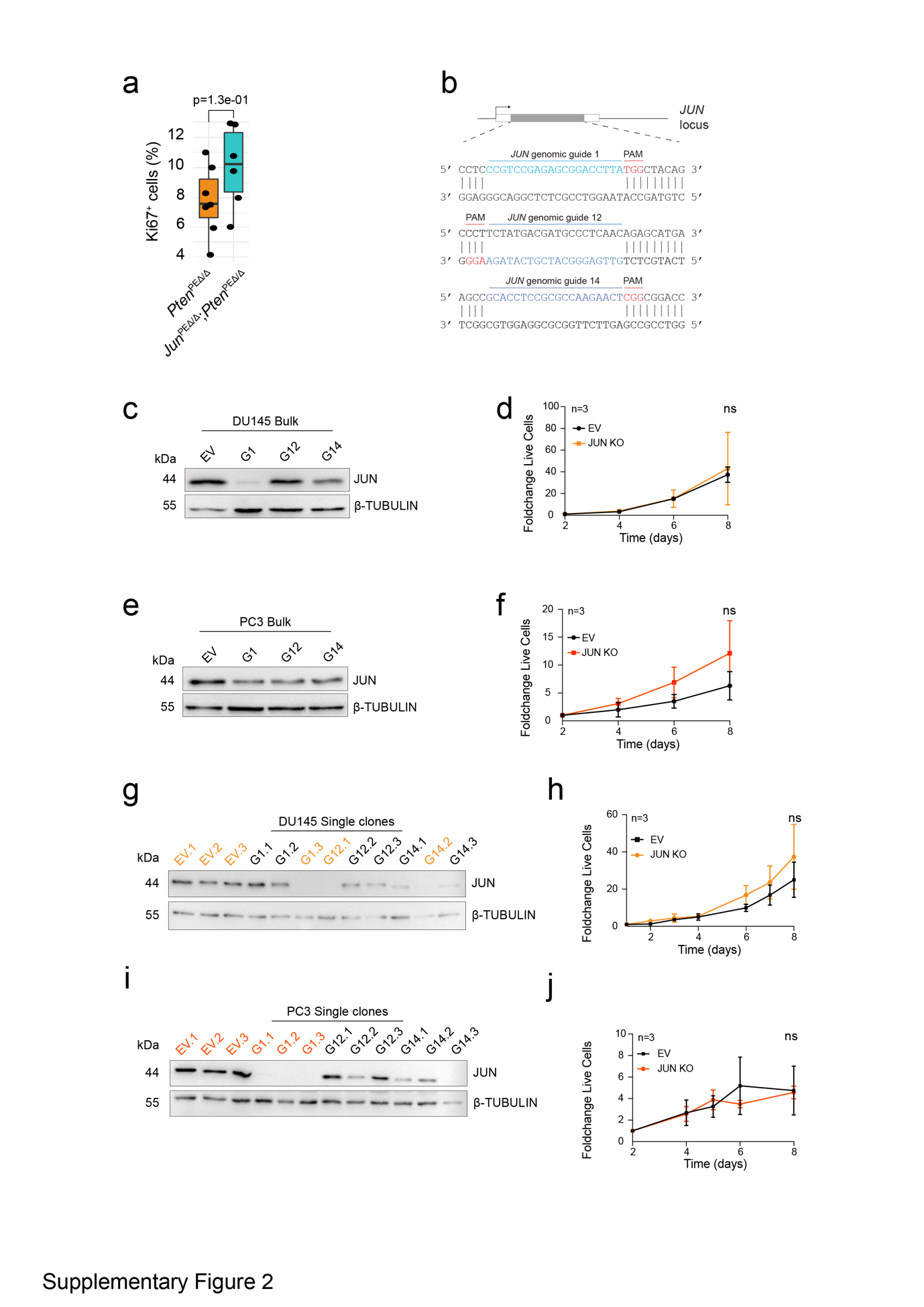


**Supplementary Figure 2: JUN does not control cellular proliferation in *Pten*-deficient prostate tumors** a) Box plot depicting the percentage of Ki67^+^ epithelial cells assessed from IHC staining of *Pten^PEΔ/Δ^* (n=7) and *Jun^PEΔ/Δ^;Pten^PEΔ/Δ^* (n=6) prostate samples. Data points represent the mean of four individual regions of interest with a radius of 150 µm analyzed by manually counted positive epithelial cells. b) Schematic representation of three individual CRISPR guide RNAs designed to target the genomic human *JUN* locus. The sequence of the guide RNA is depicted in blue and the protospacer adjacent motif (PAM) in red. c) Western blot showing protein levels of JUN in bulk culture DU145 PCa cell lines subjected to CRISPR/Cas9 deletion of *JUN* (G1, G12, G14) and empty vector transduced control (EV). β-Tubulin serves as loading control. The molecular weight of analyzed proteins in kiloDaltons (KDa) is shown on the left. d) Viable cell count-based proliferation curve of DU145 bulk cultures shown in c). Cell density of first measurement is set to 1 and density measure on consecutive days is calculated as fold-changes. Each measurement contains three biological replicates (n=3) of empty vector transduced (EV) and CRISPR guide transduced (JUN KO). A two-sided unpaired t-test revealed no statistical significance (ns). e) As in c) but using PC3 human PCa cell lines. f) As in d) but using PC3 human PCa cell lines. g) Western blot showing protein levels of JUN in DU145 single clones picked following CRISPR/Cas9 deletion of *JUN* (G1, G12, G14) and empty vector transduced control (EV). β-Tubulin serves as loading control. The molecular weight of analyzed proteins in kiloDaltons (KDa) is shown on the left. The single clones marked in orange were used as biological replicates for the proliferation curve in h). i) As in d, f) but using DU145 single clones with robust loss of JUN protein. i) As in g) using PC3 single clones. j) As in h) using PC3 single clones in three biological replicates.


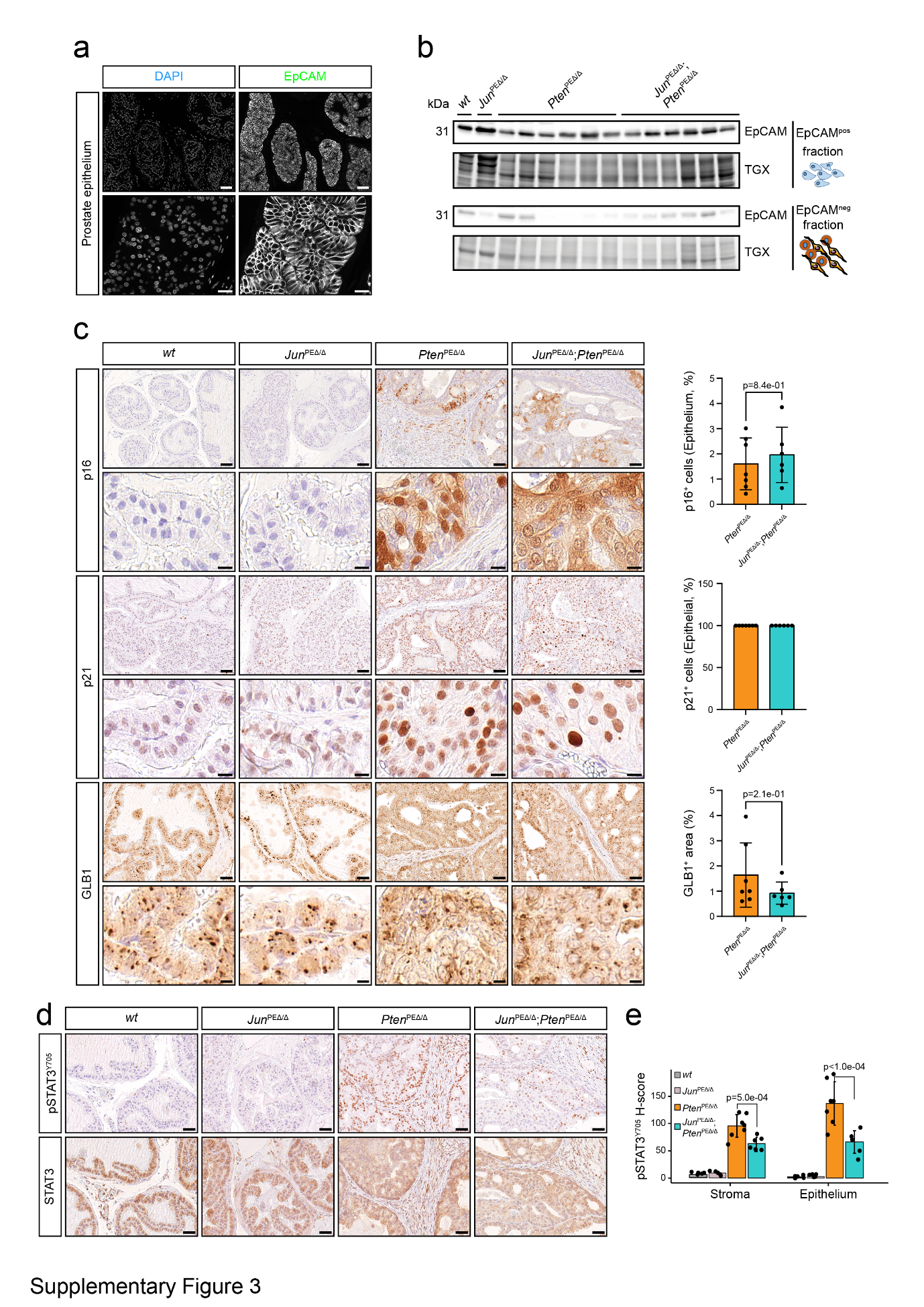


**Supplementary Figure 3: EpCAM expression in epithelial and stromal cells and analysis of cellular senescence markers.** A) Single channel grayscale representative images of DAPI/EpCAM immunofluorescence (IF). Top row: 40.0x magnification, scale bar represents 60 µm; Bottom row: 147.5 x magnification, scale bar represents 20 µm. b) Immunoblot analysis with an antibody against EpCAM to survey separation of mouse prostate sample single cell suspensions in EpCAM positive and negative fractions after magnetic cell sorting in all experimental groups. TGX stain free technology is used as loading control. c) Representative pictures of IHC stainings and corresponding quantifications with antibodies against p16^INK4A^, p21^CIP1/WAF1^ and GLB1. First and third row: 40.0x magnification, scale bar represents 60 µm; Second and fourth row: 300.0 x magnification, scale bar represents 10 µm. Fifth row: 63.0x magnification, scalebar represents 40 µm. Sixth row: 400.0x magnification, scalebar represents 6 µm. Statistical significance between *Pten^PE^*^Δ/Δ^ and *Jun^PEΔ/Δ^;Pten^PEΔ/Δ^* animals was assessed with an unpaired Students t-test. d) Representative pictures of IHC stainings with antibodies against phosphorylated STAT3 at tyrosine Y705 (pSTAT3^Y705^) (upper panels) and total STAT3 (lower panels) in all four experimental genotypes. Images were taken in a 63.0x magnification and the scale bar represents 40 µm. e) Quantification of IHC staining with an antibody against pSTAT3^Y705^ in indicated biological replicates of 19-week-old *wt*, *Pten^PE^*^Δ/Δ^, *Jun^PEΔ/Δ^* and *Jun^PEΔ/Δ^;Pten^PEΔ/Δ^* animals according to H-score. Stroma and epithelium were analysed individually by digital pathology software on whole slide scans. Statistical testing was done with one-way Anova with Turkey’s multiple comparison and statistical significance between *Pten^PE^*^Δ/Δ^ and *Jun^PEΔ/Δ^;Pten^PEΔ/Δ^* animals is shown


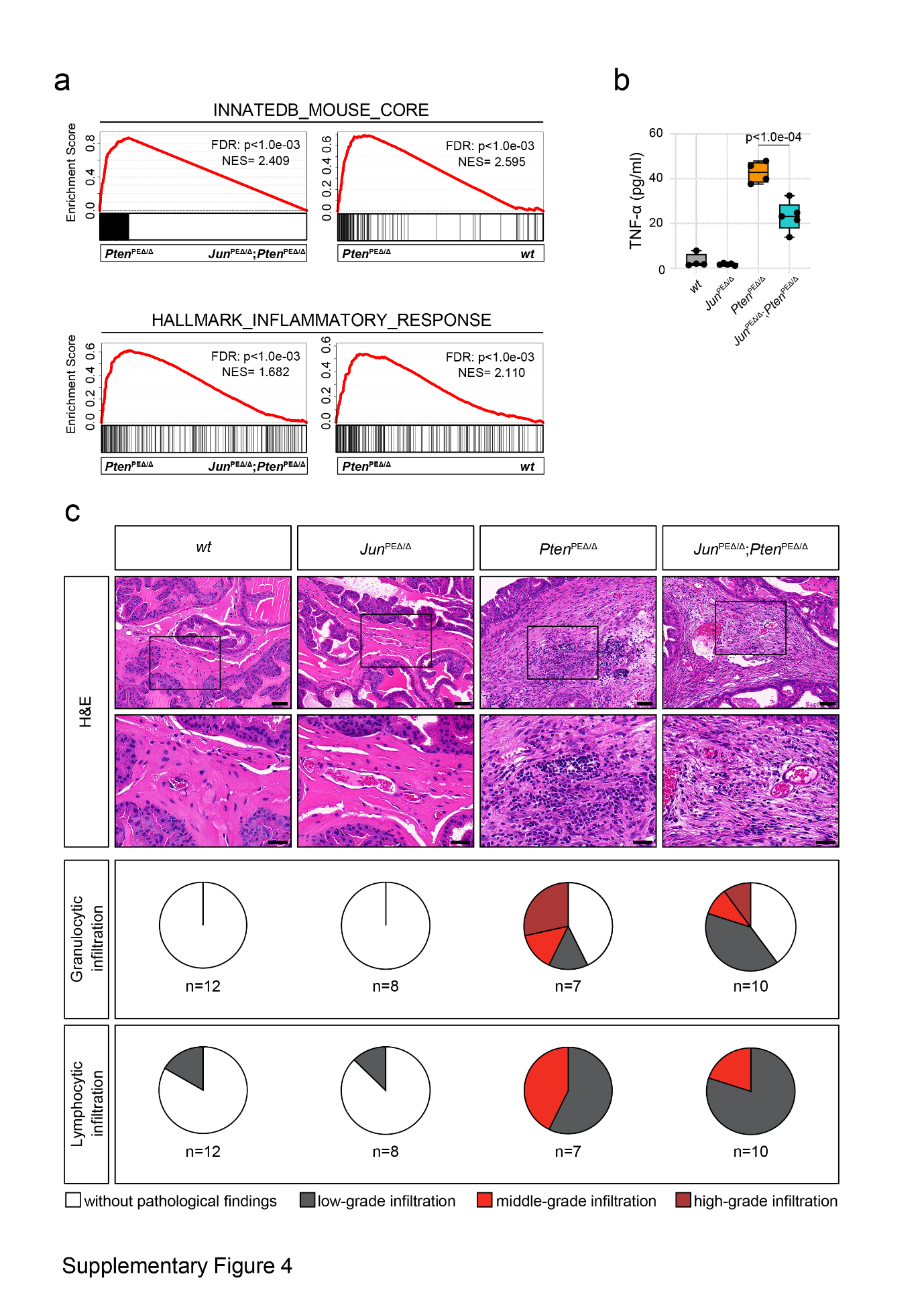


**Supplementary Figure 4: JUN level determines stages of immune cell infiltration.** a) GSEA enrichment analysis using InnateDB_mouse_core (upper panels) and HALLMARK_INFLAMMATORY_RESPONSE gene sets in *Pten^PE^*^Δ/Δ^ versus *Jun^PEΔ/Δ^;Pten^PEΔ/Δ^* and *Pten^PE^*^Δ/Δ^ versus *wt* animals. b) Multiplex immunoassay of homogenized prostate samples of 19-week-old *wt*, *Jun^PEΔ/Δ^, Pten^PE^*^Δ/Δ^ and *Jun^PEΔ/Δ^;Pten^PEΔ/Δ^* animals for analysis of TNF-α levels in pico grams (pg)/ml of indicated biological replicates. Statistical testing was done with unpaired t-test, p-value between *Pten^PEΔ/Δ^* and *Jun^PEΔ/Δ^;Pten^PEΔ/Δ^* groups is indicated. c) Upper panels: Representative H&E images depicting the morphology of prostates and infiltration of granulocytes and lymphocytes in 19-week-old animals. Top row: 40.0x magnification, scale bar represents 60 µm; Bottom row: 100.0 x magnification, scale bar represents 30 µm. Lower panels: The sections were evaluated by an independent pathologist and the level of immune cell infiltration (white = without infiltration; grey = low-grade; red = middle-grade; dark red = high-grade infiltration) of *wt* (n=12), *Jun^PEΔ/Δ^* (n=8), *Pten^PEΔ/Δ^* (n=7) and *Jun^PEΔ/Δ^;Pten^PEΔ/Δ^* (n=10) prostates were assessed and summarized in pie charts.


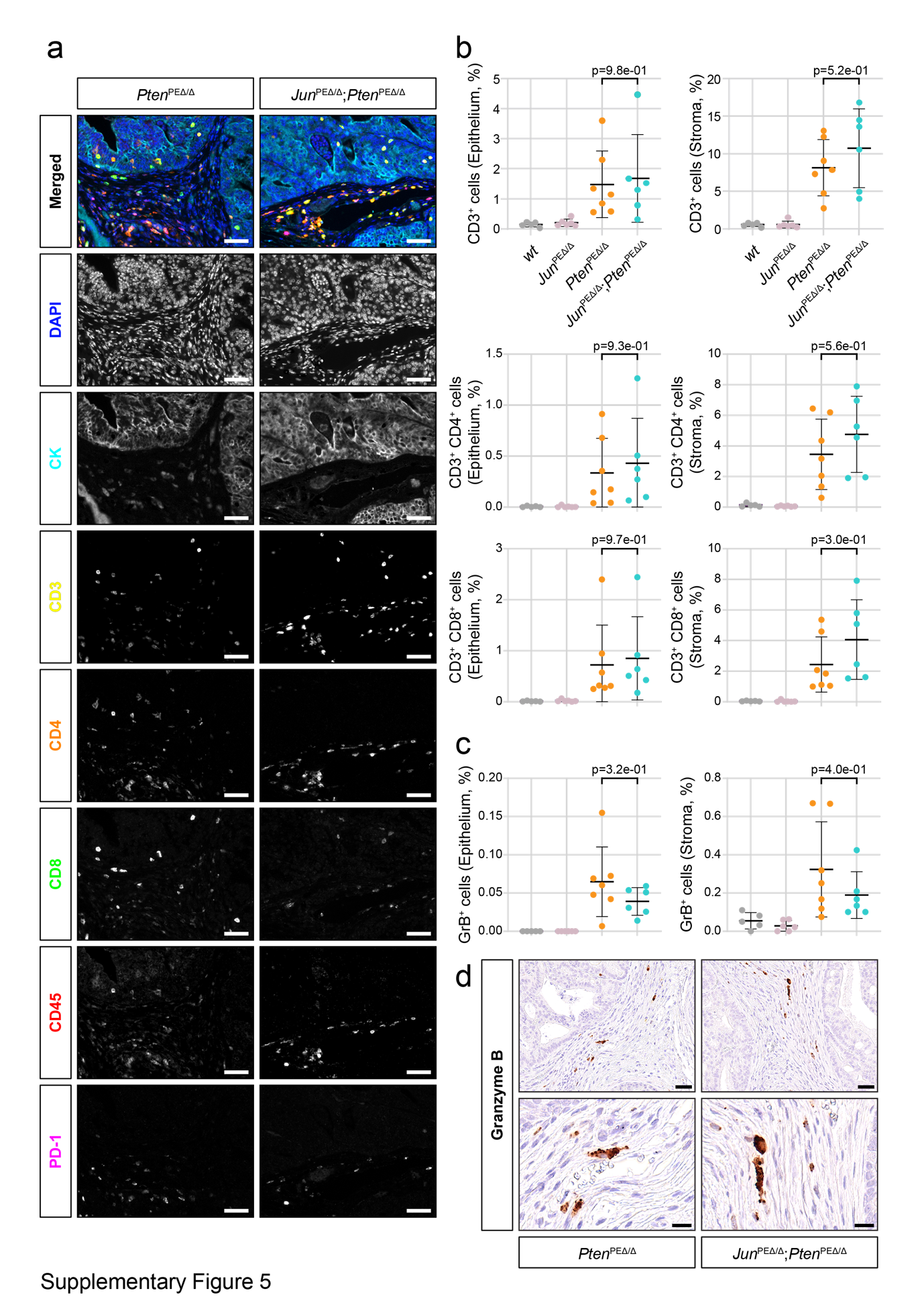


**Supplementary Figure 5: The composition of T cell subsets is not affected by *Jun*-deficiency.** a) Merged and single channel grayscale representative images of multiplex IHC of sections of *Pten^PEΔ/Δ^* and *Jun^PEΔ/Δ^;Pten^PEΔ/Δ^* prostates for assessment of immune cell subsets. Panel cell surface markers: cytokeratin (CK)/CD3/CD4/CD8/CD45/PD-1/DAPI. Scale bars represent 50µm. b) Quantification of tumor (left) or stroma (right) infiltrating CD3^+^, CD3^+^ CD4^+^ and CD3^+^ CD8^+^ T cells reveals no statistical difference. c) Quantification of Granzyme B IHC staining shows no significant difference between *Pten^PEΔ/Δ^* and *Jun^PEΔ/Δ^;Pten^PEΔ/Δ^* prostates. d) Representative images of Granzyme B IHC staining. Top row: 63.0x magnification, scale bar represents 40 µm; Bottom row: 200.0x magnification, scale bar represents 15 µm.
