# Supplementary material for: JUN mediates the senescence associated secretory phenotype and immune cell recruitment to prevent prostate cancer progression
Source: Mol Cancer. 2024 May 29;23:114. doi: 10.1186/s12943-024-02022-x (PMC11134959; doi:10.1186/s12943-024-02022-x)
Supplement: Supplementary file 3 — Supplementary Material 3. [file 12943_2024_2022_MOESM3_ESM.docx]

**Supplementary Materials and Methods**

**Cell culture**

HEK293FT [1], DU145 [2] and PC3 [3] cell lines were used for the experiments. HEK293FT cells were cultivated in Dulbecco’s Modified Eagle Medium (DMEM) supplemented with 10% Fetal Bovine Serum, 1 U/ml of Penicillin-Streptomycin solution and 2mM L-Glutamine (all Thermo Fisher Scientific). PC3 and DU145 cell lines were cultivated in Roswell Park Memorial Institute (RPMI) medium supplemented with 10% Fetal Bovine Serum, 1 U/ml of Penicillin-Streptomycin solution, 2mM L-Glutamine and 2.5mM HEPES (all Thermo Fisher Scientific).

**Constructs, lentiviral particle production and cell transduction**

# *CRISPR/Cas9 Genome Editing:* CRISPR guide RNAs targeting exon 1 of human *JUN* (ENST00000371222.3) were designed with the webtool previously available at [www.crispr.mit.edu](http://www.crispr.mit.edu). The guide RNA sequences in a 5’ to 3’ orientation were G1: CCGTCCGAGAGCGGACCTTA; G12: GTTGAGGGCATCGTCATAGA; G14: GCACCTCCGCGCCAAGAACT. The desalted oligonucleotides including *BsmBI* restriction sites were obtained from Microsynth. Guide RNAs were cloned into lentiCRISPR v2 vector obtained from Addgene. LentiCRISPR v2 was a gift from Feng Zhang (Addgene plasmid #52961). For cloning, the Zhang lab protocol available at Addgene was followed [4, 5]. The guide RNAs were tested for cutting efficiency with a T7 Endonuclease I assay (NEB) as previously described [6] and the CRISPR cutting efficacy was determined with the TIDE webtool [7].

# *Virus production:* Generation of viral supernatants was done as previously described [8, 9]. In brief, lentiviral particles were produced by co-transfecting constructs with packaging and envelope plasmids psPAX2 and pMD2.G in HEK293FT cells, seeded in 10 cm tissue culture dishes using Lipofectamine 2000 (Life Technologies). psPAX2 was a gift from Didier Trono (Addgene plasmid #12260). pMD2.G was a gift from Didier Trono (Addgene plasmid #12259). 72 hours post-transfection, viral supernatants were harvested and virus was concentrated using the PEG virus precipitation kit (BioVision, Inc), divided into aliquots, snap-frozen in liquid nitrogen and stored at -80°C.

# *Transduction and selection of bulk culture:* 2.5x10^5^ DU145 and PC3 cells were transduced with 30 μl of viral supernatant in the presence of 10 μg/ml hexadimethrine bromide (Sigma) in 6-well plates for 72 hours. Cells were selected with 2 μg/ml puromycin (DU145) and 2.5 μg/ml puromycin (PC3) for 48 hours.

# Single clone picking

# Single clones were established by passaging transduced cell lines in a dilution rate of 1:1000 to 1:5000 on 10cm dishes. When single clones became macroscopically visible, plates were washed with 1x PBS and individual clones were detached by pipetting 20 μl of trypsin directly on the colony and repeatedly pipetted up and down. The clones were then transferred to a 24-well plate and grown to confluency.

# Cellular Proliferation analysis

# To perform cell count-based proliferation analysis, we used the LUNA-II Automated Cell Counter in three replicate cell lines per genotype. 2.5x10^4^ viable DU145 and 9x10^4^ PC3 cells were pipetted into a 6-well plate for each replicate. The cell density was measured at multiple timepoints (Day2, 4, 6 and 8). The density measurements of the first timepoint were set to 1 and later timepoints were calculated as fold-change.

**RNA isolation from prostate tissue**

25 mg of RNAlater (Sigma-Aldrich) treated snap-frozen prostate tissue was homogenized in TRI reagent (Merck) with a T10 homogenizer (IKA) according to manufacturer’s instructions. The aqueous, RNA containing phase mixed with 70% EtOH was transferred to a ReliaPrep Minicolumn (Promega) and a DNAseI digest was performed directly on the column following the manufacturer’s protocol. The DNAseI digested, cleaned RNA was eluted in 25 μl nuclease-free H_2_O and the quality control was performed on the 4200 Tapestation (Agilent). The concentration was determined on a DS-11 FX+ nanophotometer (DeNovix).

**Gene set-enrichment analyses and expression analyses**

Gene set-enrichment analysis (GSEA) and single sample GSEA were performed using the stand-alone software tool (GSEA v4.3.2) or latest R script GSEA-R v1.2 and self-defined gene signatures or defined signatures as provided by the Broad Institute MSigDB, version 6.1.1 [10]. The “ReplotGSEA” tool (https://rdrr.io/github/PeeperLab/Rtoolbox/man/ ReplotGSEA.html#heading-4) was used for generation of high quality enrichment plots. For the validation of a senescent phenotype of PCa we applied senescence-specifying gene signatures “FRIDMAN_SENESCENCE_UP”, “REACTOME_CELLULAR_ SENESCENCE” and “REACTOME_ONCOGENE_INDUCED _SENESCENCE”. The SASP phenotype was primarily validated by signatures “REACTOME_SENESCENCE_ASSOCIATED_ SECRETORY_PHENOTYPE_SASP”, “GUCCINI_CORE_SASP_UP” [11] and SenMayo (“SAUL_SEN_MAYO”, mouse) signature [12]. In addition, we utilized the H, C2 (hallmark gene sets, human collections), MH (mouse-ortholog hallmark gene sets) and M5 (ontology gene sets) or single gene signatures (“Inflammatory response”, “Neutrophil migration/chemotaxis”). Genes represented in the “InnateDB_core” signature were derived from “InnateDB: Systems Biology of the Innate Immune Response” [13]. All gene signatures are available in Supplementary Table 5. For representation of “Top differentially regulated pathways” (Fig. 3d), we performed GSEA using H and C2 and selected the top enriched (FDR≤0.05) signaling pathways of comparisons *wt* versus *Pten^PEΔ/Δ^* and *Pten^PEΔ/Δ^* versus *Jun^PEΔ/Δ^;Pten^PEΔ/Δ^.* For gene ontology (GO)-term analysis of DEGs among *Pten^PEΔ/Δ^* versus *Jun^PEΔ/Δ^;Pten^PEΔ/Δ^* (Fig. 3h), we used signature M5. The ggplot2 R package was used for heat map and bubble chart-based representation.

**Statistical analysis of RNA sequencing data**

Box plots represent data of n≥3 samples (stated) and show median (center line), the upper and lower quartiles (the box), and the range of the data (the whiskers), including outliers. Significance was determined by an unpaired, two-sided t-test using R. Kaplan-Meier survival plots using the KMplot tool (<http://kmplot.com/private/>) were determined by cox regression analysis and statistical significance (p-value) was calculated by logrank test. We performed survival analysis using the SurvExpress webtool [14] as previously described [15]. Generally, the significance level of differences between groups was determined by two-tailed unpaired Student’s t-tests for two groups or ordinary one-way ANOVA. In GSEA analyses, only processes with p-values corrected for multiple testing (FDR-adjusted p-value <0.05) were considered significantly regulated. Expression data as determined by transcriptome profiling are represented as bar graphs with individual data points.

**Scanning**

Stained slides were scanned with a PANNORAMIC Scan II from 3DHISTECH, using the following parameters: Objective type: 20x; Output resolution: 49x native; Multilayer mode: extended focus, 7 levels, step size 1 µm; Compression: JPG; Bit depth: 8-bit; stitching enabled; and saved as MRXS files. Representative pictures for figures were exported using the snapshot function of CaseViewer (Build 2.4.0.119028).

**Multiplex immunohistochemistry**

Mouse prostate samples were stained with multiplex IHC and analysed by multispectral imaging. A panel of 6 fluorescent markers plus DAPI as a nuclear stain were used to detect the epitopes of CD3 (Abcam, clone SP162), CD4 (Abcam, clone EPR19514) CD8 (Cell Signaling, clone D4W2Z), CD45 (Abcam, clone EPR20033), PD-1 (Cell Signaling, clone D7D5W) and pan-Cytokeratin (Agilent Technologies, clone AE1/AE3). The staining of all slides was performed with autostainer system Bond RX (Leica Biosystems Inc.). The slides were then scanned with the Vectra® 3 (Akoya Biosystems; software version 3.0.7) microscope. Whole-slide scans were taken at 4x magnification to define regions of interest (whole tissue area) to be scanned in higher resolution using Phenochart software, version 1.0.12. Multispectral images of defined areas (whole tissue) were recorded with 20x magnification, resulting in one image color channel for each stained antibody. Images were processed with inForm software (Akoya Biosystems; software version 2.4.10), including spectral unmixing and removal of autofluorescence. Multispectral images were evaluated using HALO® Image Analysis Platform (Indica Labs). Single recorded images at 20x magnification are stitched together into a continuous field of view of the whole tissue. Individual cells were then identified using the DAPI nucleus staining by setting a threshold for nucleus size, roundness and signal intensity. For the 6 fluorescently labeled markers, positivity thresholds were set according to the staining intensity.

**Metastases Analysis**

H&E prostate tissue sections from heart, liver, lung, kidneys, spleen, lymph nodes and brains of 39 week old *wt* (n=6)*, Jun^PEΔ/Δ^* (=5)*, Pten^PEΔ/Δ^* (=5) *and Jun^PEΔ/Δ^;Pten^PEΔ/Δ^*(=5) animals were examined for metastatic lesion formation by two independent pathologists.

**Luminex cytokine array**

Snap frozen prostate tissue was homogenized in 500 µl Schindler’s lysis buffer [16] using a 1600 MiniG® tissue homogenizer and steel beads for 60 seconds at 1500 rpm. The homogenized solution was centrifuged for 30 minutes at 21000 xg and 4°C, after which the supernatant was transferred and measured for its protein concentration. 100 µg of protein were used to measure cytokine concentration using the ProcartaPlex Mouse Basic Kit (Invitrogen™, EPX010-20440-901) in combination with the respective simplex kits for each cytokine (Invitrogen™, EPX01A-26002-901, EPX01A-20603-901, EPX01A-20607-901). Immunoassays were performed according to the manufacturer’s instructions and measured with a Bio-Plex 200 (Bio-RAD) system. Significance was determined using an ordinary one-way ANOVA with Tukey’s multiple comparisons tests for three or more groups. Graphs were created and formatted in GraphPad PRISM (version 9.5.0).

**References**

1. DuBridge RB, Tang P, Hsia HC, Leong PM, Miller JH, Calos MP (1987) Analysis of mutation in human cells by using an Epstein-Barr virus shuttle system. Mol Cell Biol 7:379–87

2. Stone KR, Mickey DD, Wunderli H, Mickey GH, Paulson DF (1978) Isolation of a human prostate carcinoma cell line (DU 145). Int J Cancer 21:274–81

3. Kaighn ME, Narayan KS, Ohnuki Y, Lechner JF, Jones LW (1979) Establishment and characterization of a human prostatic carcinoma cell line (PC-3). Invest Urol 17:16–23

4. Shalem O, Sanjana NE, Hartenian E, et al (2014) Genome-scale CRISPR-Cas9 knockout screening in human cells. Science 343:84–87

5. Sanjana NE, Shalem O, Zhang F (2014) Improved vectors and genome-wide libraries for CRISPR screening. Nature Publishing Group 11:783–784

6. Shah RR, Cholewa-Waclaw J, Davies FCJJ, Paton KM, Chaligne R, Heard E, Abbott CM, Bird AP (2016) Efficient and versatile CRISPR engineering of human neurons in culture to model neurological disorders. Wellcome Open Res 1:13

7. Brinkman EK, Chen T, Amendola M, van Steensel B (2014) Easy quantitative assessment of genome editing by sequence trace decomposition. Nucleic Acids Res 42:e168–e168

8. Lagger S, Meunier D, Mikula M, et al (2010) Crucial function of histone deacetylase 1 for differentiation of teratomas in mice and humans. EMBO J 29:3992–4007

9. Garces de los Fayos Alonso I, Zujo L, Wiest I, et al (2022) PDGFRβ promotes oncogenic progression via STAT3/STAT5 hyperactivation in anaplastic large cell lymphoma. Mol Cancer. https://doi.org/10.1186/s12943-022-01640-7

10. Subramanian A, Tamayo P, Mootha VK, et al (2005) Gene set enrichment analysis: A knowledge-based approach for interpreting genome-wide expression profiles. Proceedings of the National Academy of Sciences 102:15545–15550

11. Guccini I, Revandkar A, D’Ambrosio M, et al (2021) Senescence Reprogramming by TIMP1 Deficiency Promotes Prostate Cancer Metastasis. Cancer Cell 39:68-82.e9

12. Saul D, Kosinsky RL, Atkinson EJ, et al (2022) A new gene set identifies senescent cells and predicts senescence-associated pathways across tissues. Nat Commun. https://doi.org/10.1038/s41467-022-32552-1

13. Breuer K, Foroushani AK, Laird MR, Chen C, Sribnaia A, Lo R, Winsor GL, Hancock REW, Brinkman FSL, Lynn DJ InnateDB: systems biology of innate immunity and beyond-recent updates and continuing curation. https://doi.org/10.1093/nar/gks1147

14. Aguirre-Gamboa R, Gomez-Rueda H, Martínez-Ledesma E, Martínez-Torteya A, Chacolla-Huaringa R, Rodriguez-Barrientos A, Tamez-Peña JG, Treviño V (2013) SurvExpress: An Online Biomarker Validation Tool and Database for Cancer Gene Expression Data Using Survival Analysis. PLoS One 8:1–9

15. Oberhuber M, Pecoraro M, Rusz M, et al (2020) *STAT 3* ‐dependent analysis reveals *PDK 4* as independent predictor of recurrence in prostate cancer. Mol Syst Biol. https://doi.org/10.15252/msb.20199247

16. Prchal-Murphy M, Semper C, Lassnig C, et al (2012) TYK2 Kinase Activity Is Required for Functional Type I Interferon Responses In Vivo. PLoS One 7:e39141
