# Supplementary material for: JUN mediates the senescence associated secretory phenotype and immune cell recruitment to prevent prostate cancer progression
Source: Mol Cancer. 2024 May 29;23:114. doi: 10.1186/s12943-024-02022-x (PMC11134959; doi:10.1186/s12943-024-02022-x)
Supplement: Supplementary file 4 — Supplementary Material 4. [file 12943_2024_2022_MOESM4_ESM.docx]

**Additional File 4: Original Western Blot Membranes**

Protein Ladder used: Color Prestained Protein Standard, Broad Range NEB #P7719S

**Related to Figure 2b**


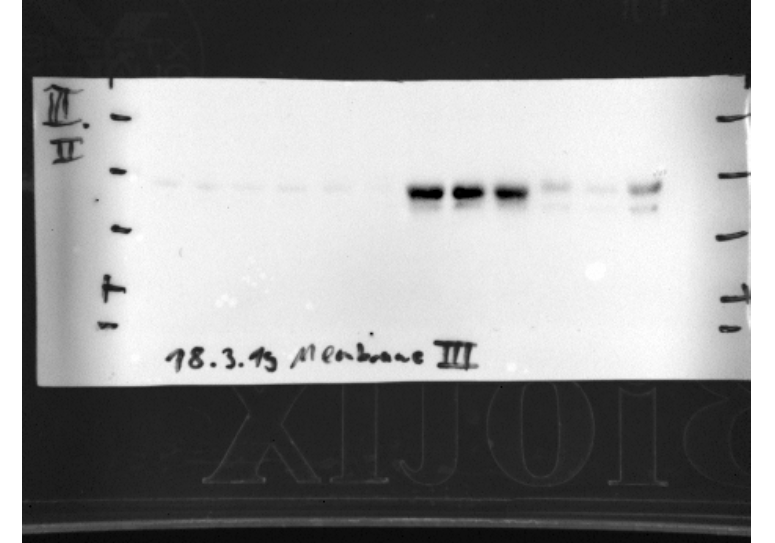
Ab: pJUN^S73^


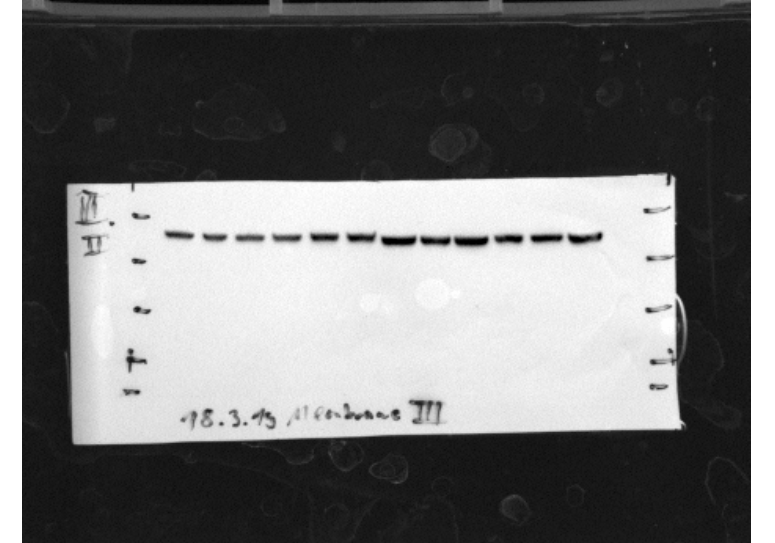
Ab: β-TUBULIN

Ab: JUN


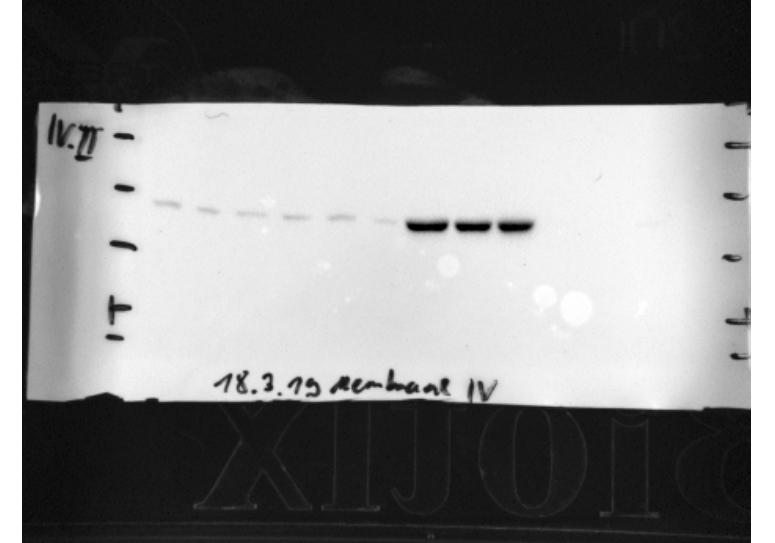


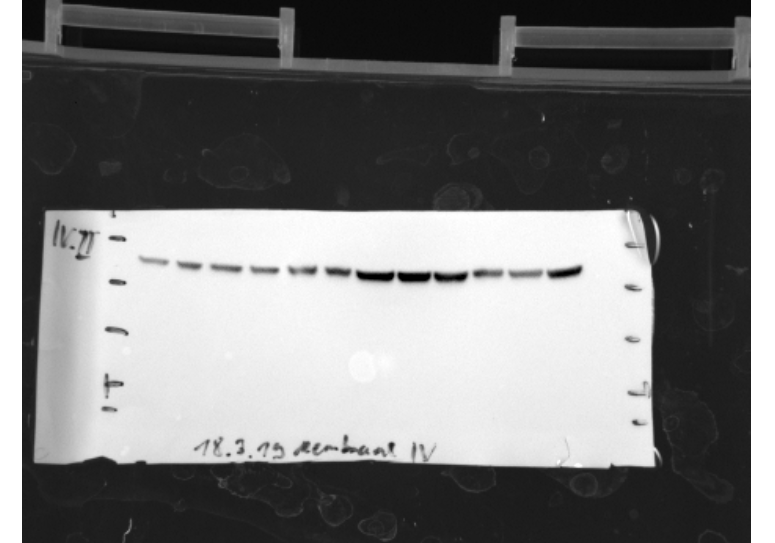
Ab: β-TUBULIN

Ab: pAKT^S473^


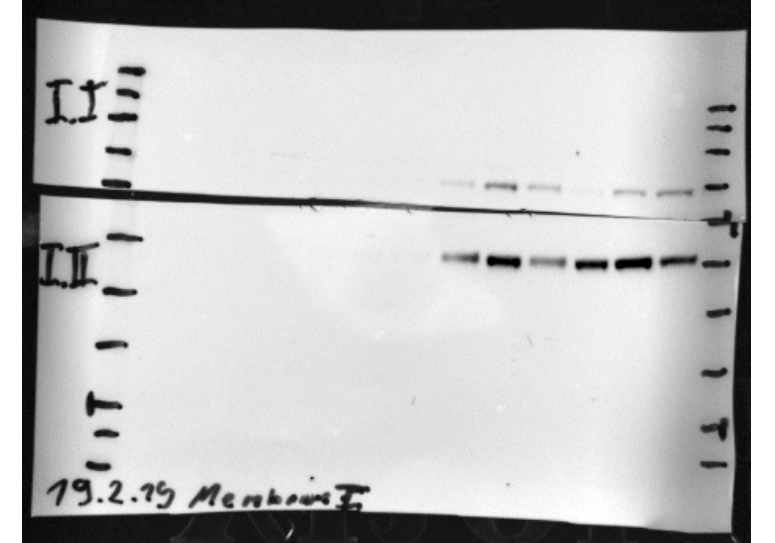


<- Unrelated Ab

<- pAKT

Ab: AKT


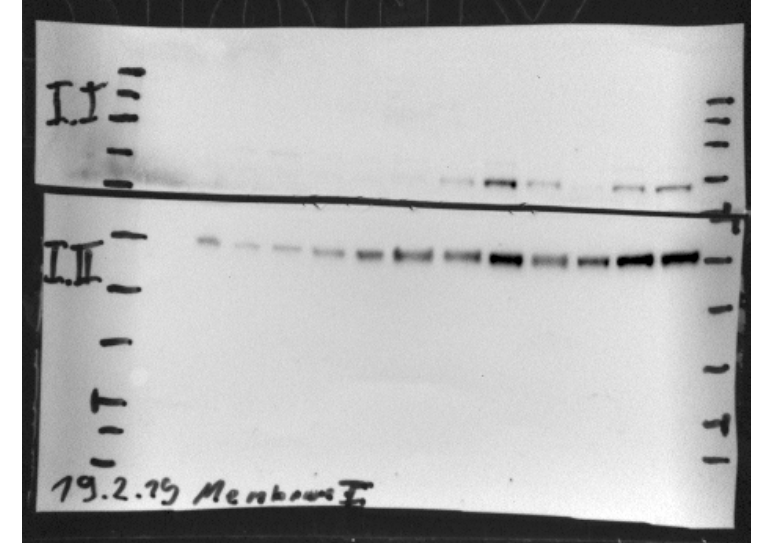


<- unrelated AB

<- AKT


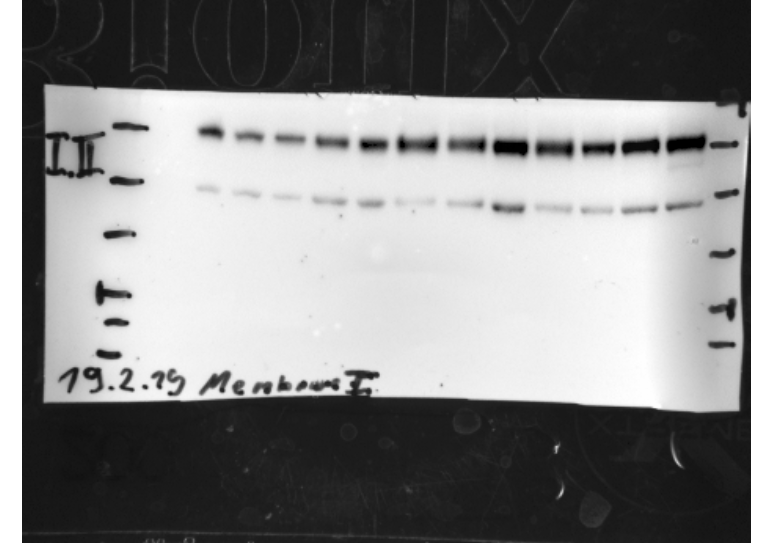
Ab: β-ACTIN

<- AKT

<- β-ACTIN

**Related to Figure 4e**

Ab: NLRP3


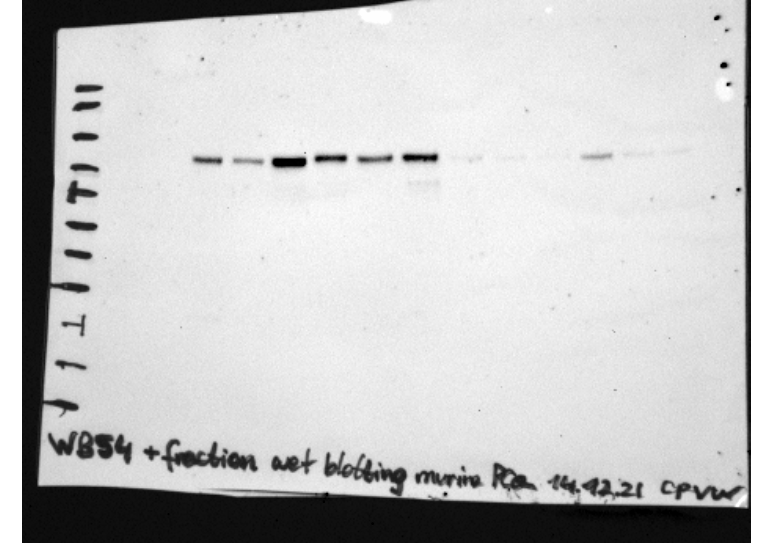


Ab: β-ACTIN


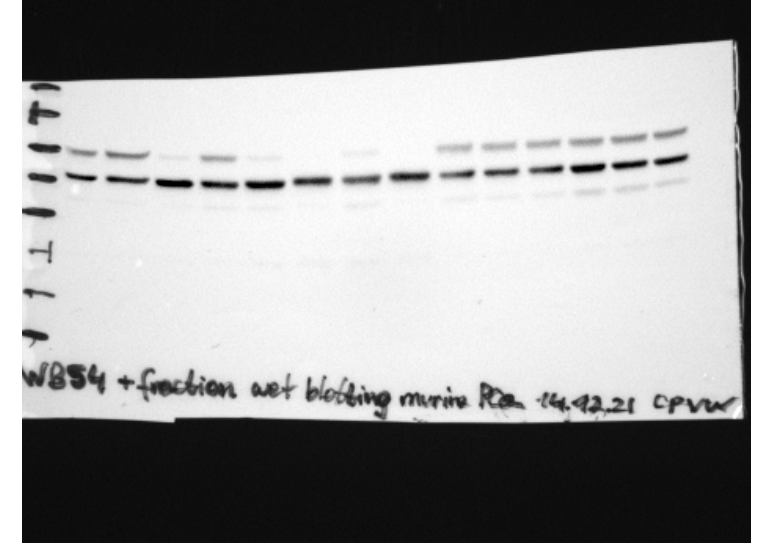


<- unspecific

Ab: Pro-IL-1β


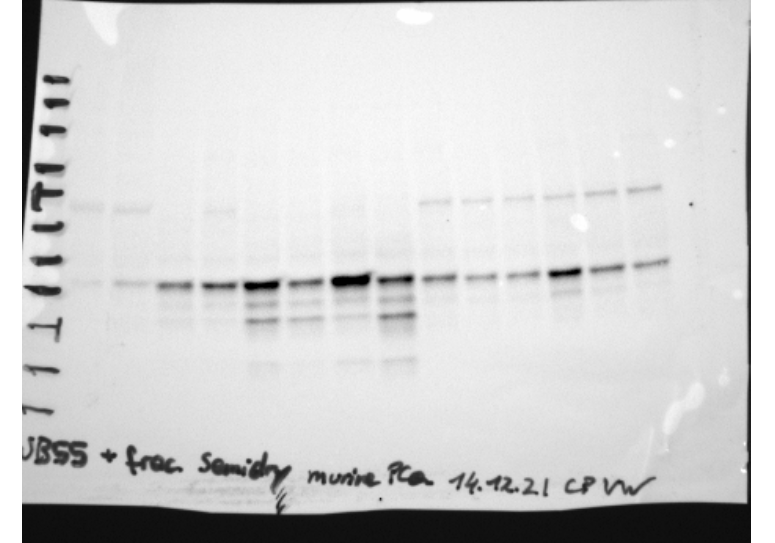


Ab: β-ACTIN


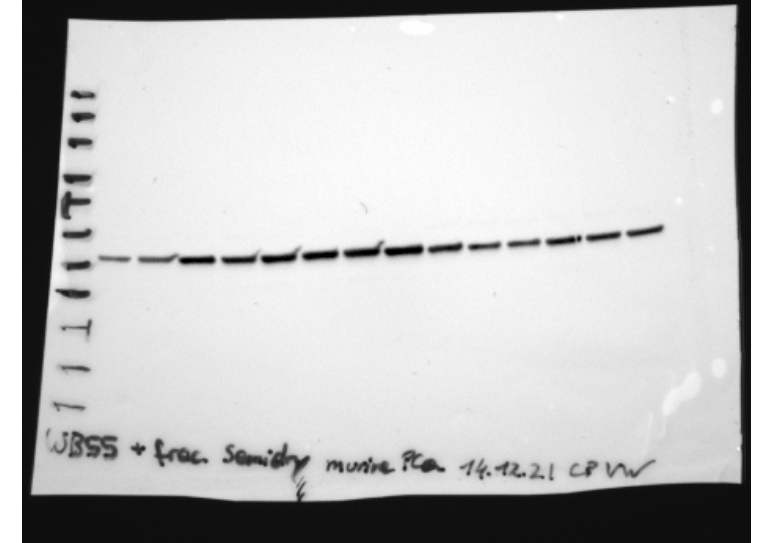


**Related to Supplementary Figure 2**

**Ab: JUN**

**
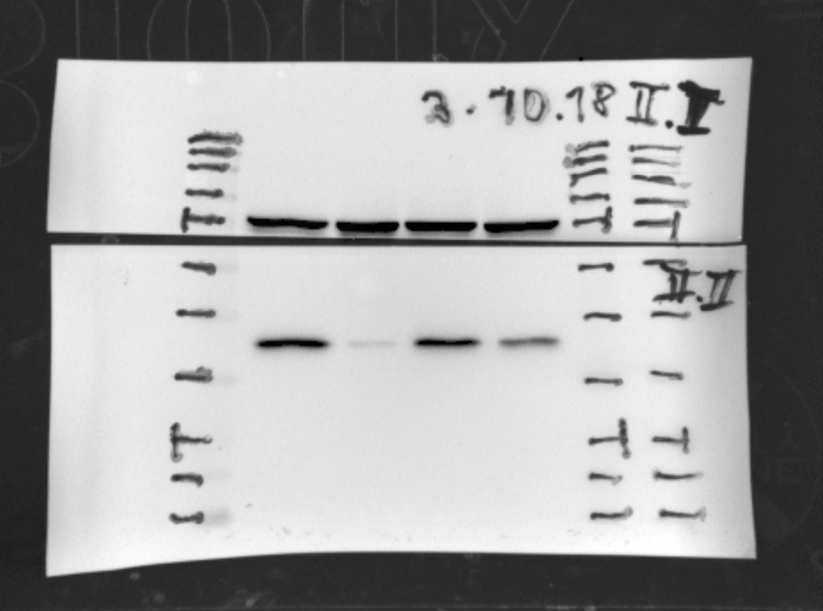
**

->STAT3 (not shown in paper)

**AB: b-Tubulin**

**
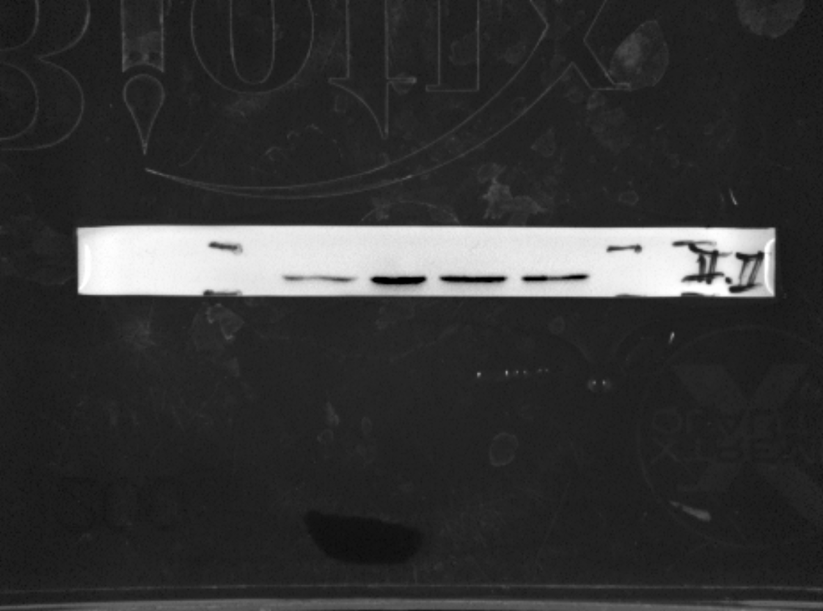
**

**AB Jun:**

**
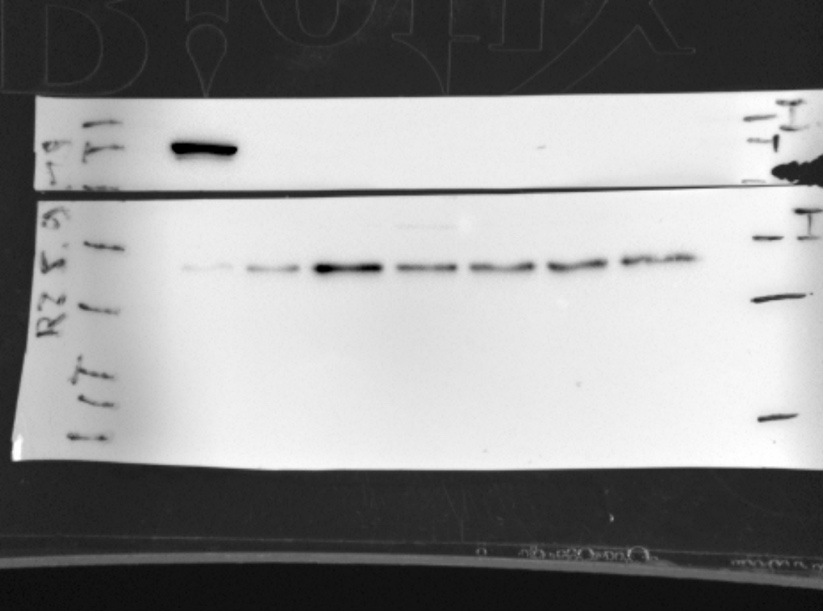
**

->STAT3 (not shown in paper)

**AB b Tubulin**

**
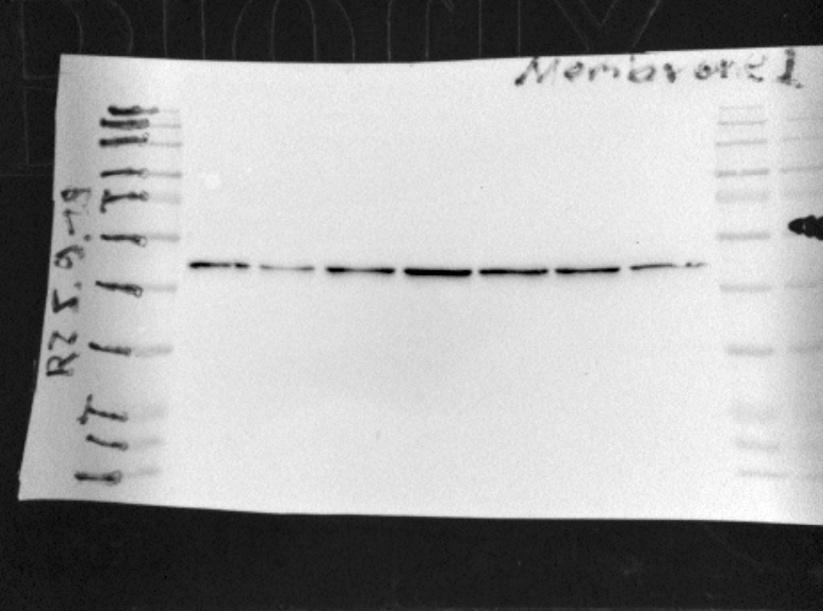
**

**Single clones AB Jun**


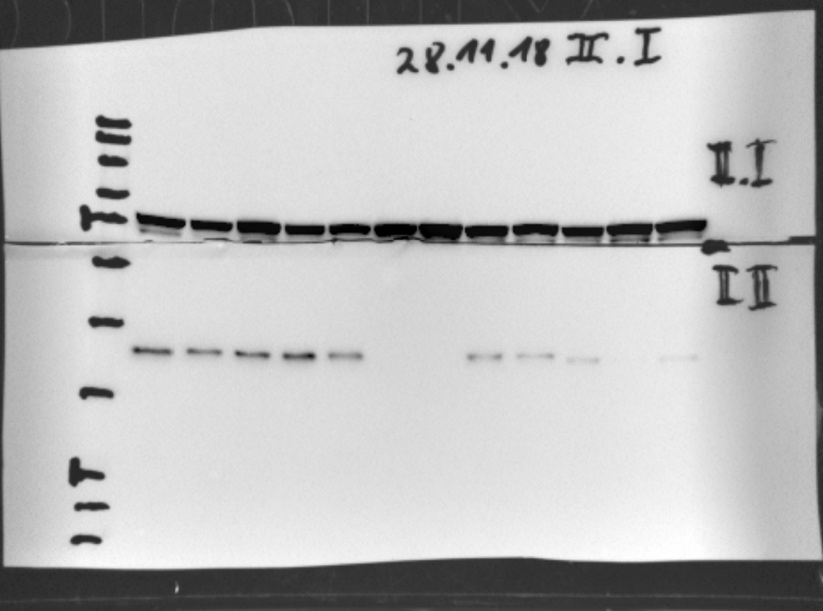


->STAT3 (not shown in paper)

**AB: b-tubulin**

**
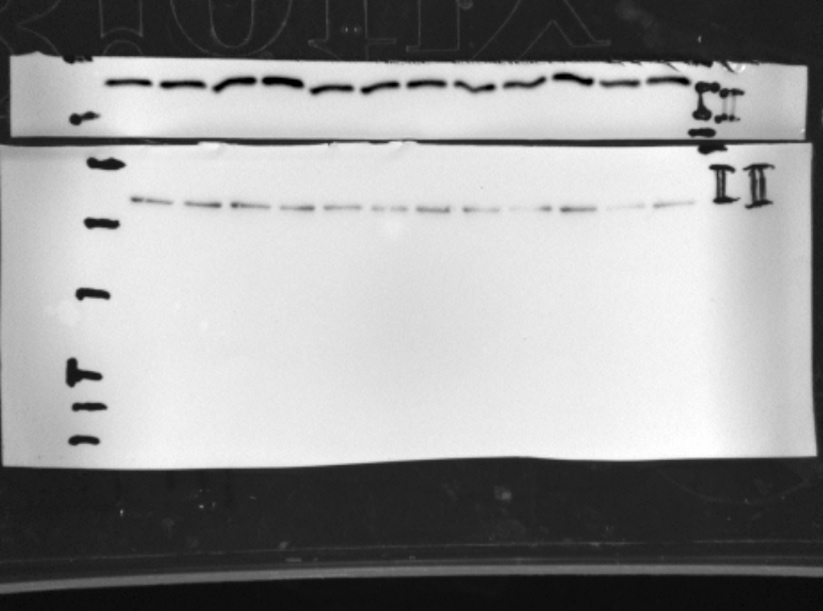
**

**AB JUN**

**
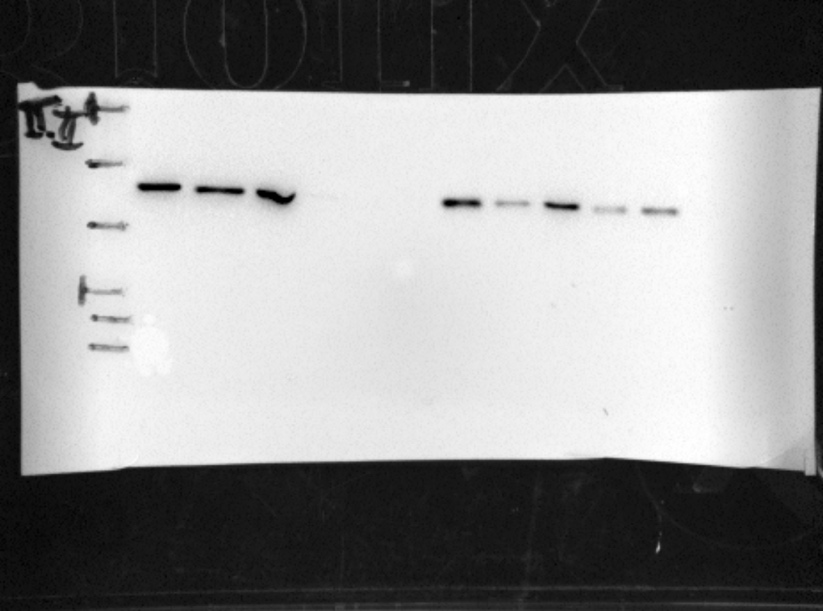
**

**AB b-Tub**

**
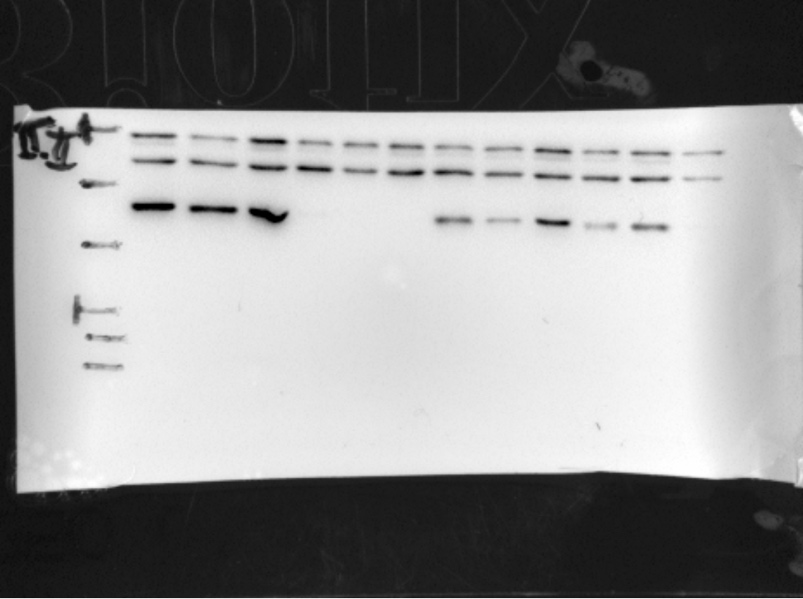
**

**Related to Supplementary Figure 3b**

Ab: EpCAM


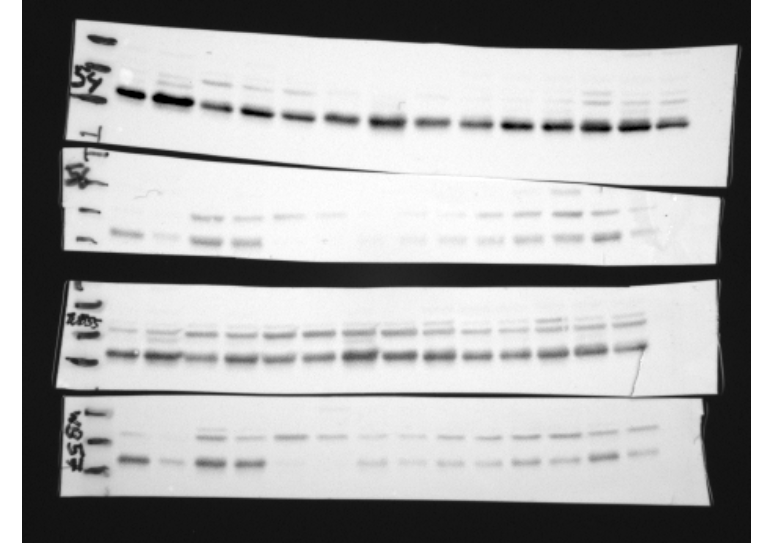


<- EpCAM positive fraction Row 1

<- EpCAM negative fraction Row 3

TGX Gel EpCAM positive fraction Row 2


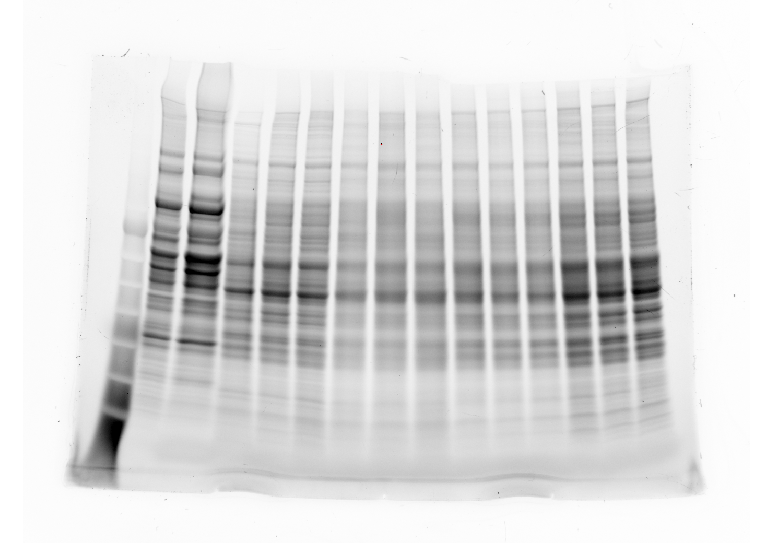


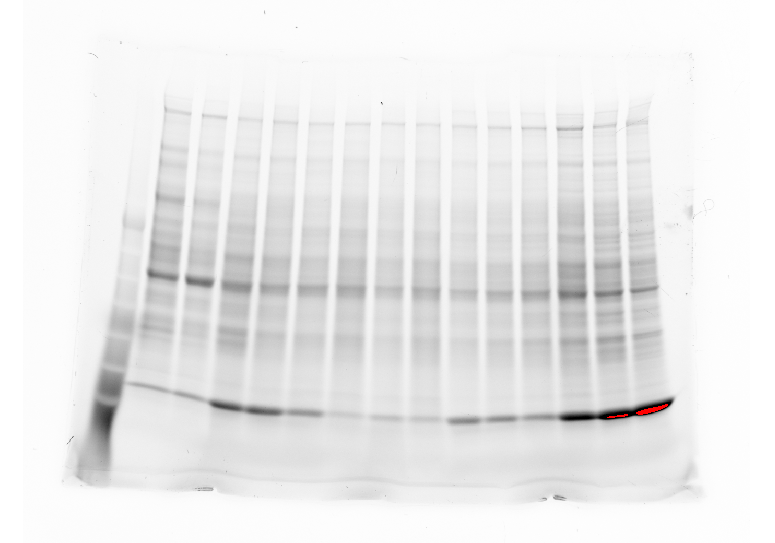
TGX Gel: EpCAM negative fraction Row 4
